# Supplementary material for: Pneumonia and Related Conditions in Critically Ill Patients—Insights from Basic and Experimental Studies
Source: Int J Mol Sci. 2022 Aug 31;23(17):9896. doi: 10.3390/ijms23179896 (PMC9456259; doi:10.3390/ijms23179896)
Supplement: Supplementary file 1 [file ijms-23-09896-s001.zip › Supplementary Figures.pdf]

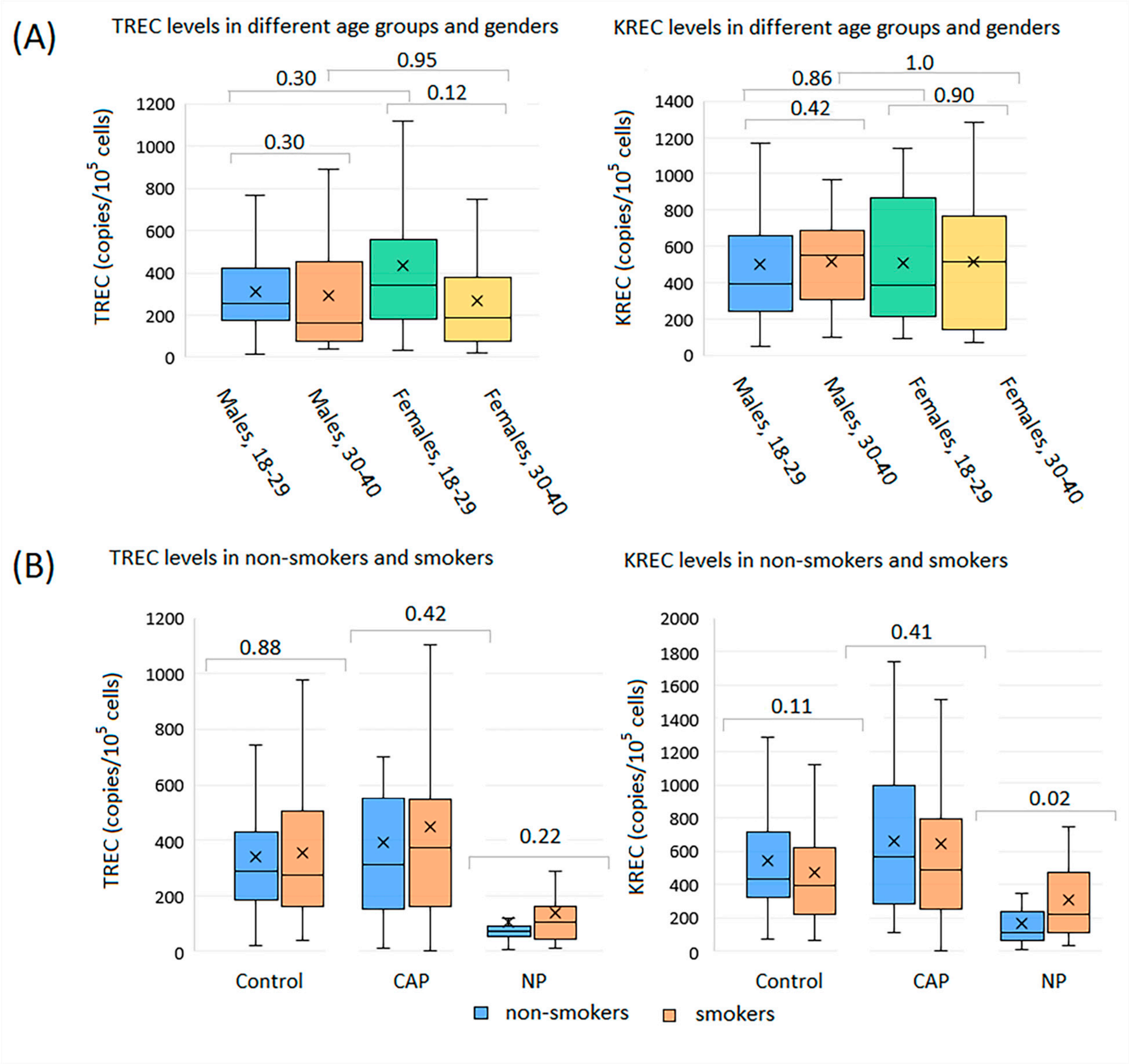

Supplementary Figure S1. The subgroup analysis of TREC/KREC counts related to sociodemographic and lifestyle factors. (A) TREC/KREC levels in the subgroups of the control group, differing in age and gender. (B) TREC/KREC levels in smokers compared to non-smokers. Abbreviations: CAP, community-acquired pneumonia; NP, nosocomial pneumonia

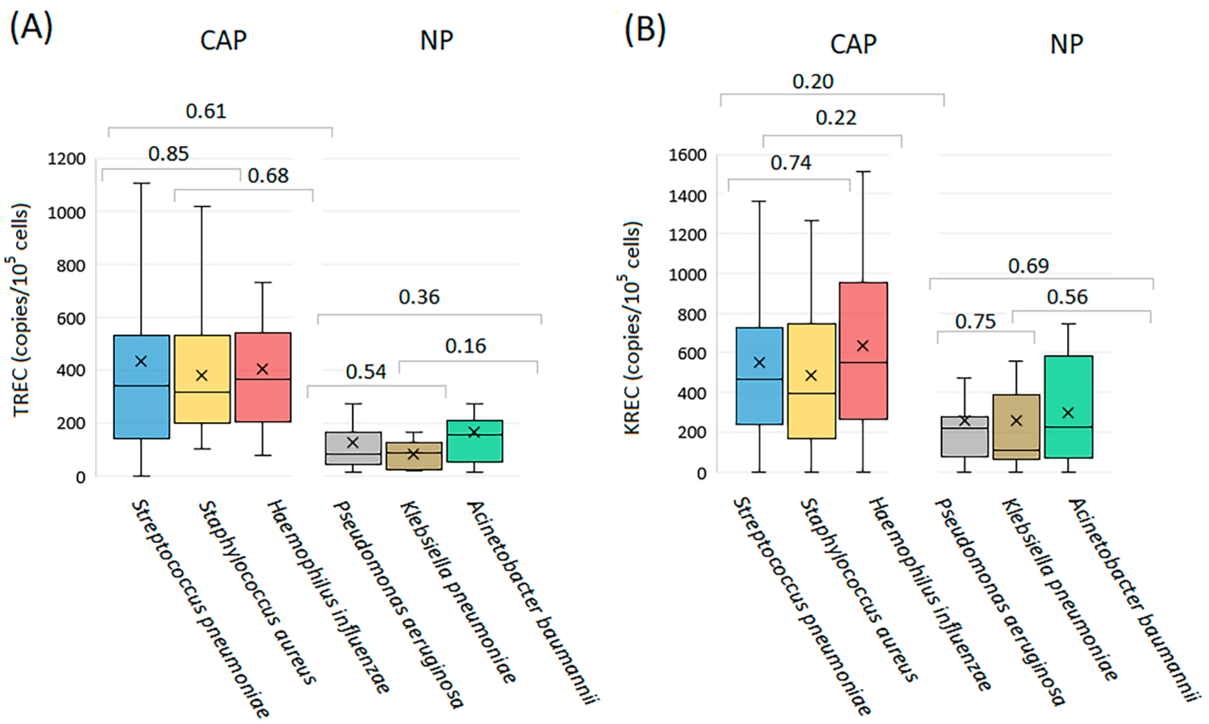

Supplementary Figure S2. The subgroup analysis of TREC (A) and KREC (B) counts in patients with pneumonia caused by certain pathogens. Abbreviations: CAP, community-acquired pneumonia; NP, nosocomial pneumonia
